# The relationship between the time of cerebral desaturation episodes and outcome in aneurysmal subarachnoid haemorrhage: a preliminary study

**DOI:** 10.1007/s10877-019-00377-x

**Published:** 2019-08-20

**Authors:** Małgorzata Burzyńska, Agnieszka Uryga, Magdalena Kasprowicz, Marek Czosnyka, Barbara Dragan, Andrzej Kübler

**Affiliations:** 1 Department of Anesthesiology and Intensive Care, Wroclaw Medical University, Wroclaw, Poland; 2 Department of Biomedical Engineering, Faculty of Fundamental Problems of Technology, Wroclaw University of Science and Technology, Wybrzeze Wyspianskiego 27, 50–370 Wroclaw, Poland; 3 Department of Clinical Neurosciences, Division of Neurosurgery, University of Cambridge, Cambridge, UK

**Keywords:** Subarachnoid haemorrhage, Near-infrared spectroscopy, Glasgow outcome scale, Hypoxia, Critical care

## Abstract

In this preliminary study we investigated the relationship between the time of cerebral desaturation episodes (CDEs), the severity of the haemorrhage, and the short-term outcome in patients with aneurysmal subarachnoid haemorrhage (aSAH). Thirty eight patents diagnosed with aneurysmal subarachnoid haemorrhage were analysed in this study. Regional cerebral oxygenation (rSO_2_) was assessed using near infrared spectroscopy (NIRS). A CDE was defined as rSO_2_ < 60% with a duration of at least 30 min. The severity of the aSAH was assessed using the Hunt and Hess scale and the short-term outcome was evaluated utilizing the Glasgow Outcome Scale. CDEs were found in 44% of the group. The total time of the CDEs and the time of the longest CDE on the contralateral side were longer in patients with severe versus moderate aSAH [h:min]: 8:15 (6:26–8:55) versus 1:24 (1:18–4:18), p = 0.038 and 2:05 (2:00–5:19) versus 0:48 (0:44–2:12), p = 0.038. The time of the longest CDE on the ipsilateral side was longer in patients with poor versus good short–term outcome [h:min]: 5:43 (3:05–9:36) versus 1:47 (0:42–2:10), p = 0.018. The logistic regression model for poor short-term outcome included median ABP, the extent of the haemorrhage in the Fisher scale and the time of the longest CDE. We have demonstrated that the time of a CDE is associated with the severity of haemorrhage and short-term outcome in aSAH patients. A NIRS measurement may provide valuable predictive information and could be considered as additional method of neuromonitoring of patients with aSAH.

**Electronic supplementary material:**

The online version of this article (10.1007/s10877-019-00377-x) contains supplementary material, which is available to authorized users.

## Introduction

Aneurysmal subarachnoid haemorrhage (aSAH) is a devastating condition that can lead to neurological disability, impairment of brain perfusion or even death. Although aSAH is not the most common type of stroke, it constitutes a burden to society since a limited number of aSAH patients are able to return to normal daily-life activities [1]. The main goal of therapy in aSAH patients is to ensure adequate brain perfusion to accomplish metabolic demand. A serious imbalance between oxygen supply and demand can lead to hypoxia, inflammation, blood–brain barrier impairment, the release of vasoconstrictors, or the development of early brain injury (EBI). In recent research, EBI has been suggested to be a major cause of further complications, disability or death in aSAH [2]. Early warning concerning a decrease in cerebral oxygenation could minimize the risk of hypoxia and EBI [3]. Monitoring of the cerebral blood flow and cerebral oxygenation can significantly improve the efficiency of intensive care and applied therapy as well as reduce the risk of poor outcome [4, 5]. Cerebral oxygenation could be monitored using invasive methods, such as brain microdialysis or thermal diffusion flowmetry [6]. The most common methods employed in clinical practise include brain tissue partial oxygen pressure (PtiO_2_) and jugular venous-oxygen saturation (SjvO_2_) [7]. Near-infrared spectroscopy (NIRS) is an alternative and promising, non-invasive technique, which continuously assesses regional cerebral saturation (rSO_2_) in bedside mode with high spatial and temporal resolution. NIRS allows for continuous, long-time monitoring of rSO_2_ in neurocritical care patients, e.g. after traumatic brain injury (TBI) [8], subarachnoid haemorrhage (aSAH) [9] or sepsis [10]. As a non-invasive method, it could be applied at an early stage of brain damage and continuously used in subsequent days after the acute phase. NIRS consists in the emission of near-infrared light (NIR) and measures the absorbance of NIR by oxy-hemoglobine (oxy-HB) and deoxy-hemoglobine (deoxy-HB) according to the Beer-Lambert law [11]. NIRS is widely applied during cardiovascular or cardiopulmonary procedures to provide information about significant decreases in cerebral oxygenation in order to prevent neurological damage or cerebral injury [12]. Moreover, it has been shown that time-resolved NIRS can predict and confirm an angiographic-proven vasospasm [13]. Few researchers have analysed the association between cerebral oxygen desaturation, based on the rSO_2_ measurements, and early and late cognitive decline or outcome in aSAH patients. Previous studies have focused either on the effectiveness of neuromonitoring using NIRS to obtain clinically relevant information in patients with cerebral vasospasm and delayed cerebral ischemia [4, 13] or on the possibility to monitor cerebral autoregulation using tissue oxygenation index, based on NIRS measurements [9]. In this paper we investigated the relationship between the time-related parameters of cerebral desaturation episodes, the severity of haemorrhage and short-term outcome in aSAH patients.

## Methods

### Setting and ethics

From January 2015 to December 2017, 46 patients with aSAH were hospitalized in the Intensive Care Unit of Wroclaw University Hospital. All subjects were adults (age ≥ 18 years), had an aneurysm in cerebral arteries which was confirmed to be the cause of bleeding using computer tomography (CT), magnetic resonance imaging (MRI) or conventional angiography and underwent continuous multimodal monitoring using Intensive Care Monitor system (ICM+; https://icmplus.neurosurg.cam.ac.uk). The exclusion criteria included the lack of written informed consent from participant or their legal representatives, death within the first 72 h from admission, changes in follow-up postoperative CT scan of the head, intracerebral hematoma, previously diagnosed cardiovascular disease or central nervous system disease. Eventually, the study group consisted of 38 patients with aSAH, who met the inclusion criteria.

The study complied with the Declaration of Helsinki of the World Medical Association. Each patient was assigned a study identification number and the data was anonymised prior to the analysis.

### Subjects and patient treatment

All aSAH patients who entered the study were treated in the ICU unit according to current guidelines [14]. Information was collected on hypertension, smoking or other medical risk factors (diabetes, mellitus etc.) before the ictus. Patient’s condition was assessed using the Glasgow Coma Scale (GCS), Full Outline of UnResponsiveness scale (FOUR) and Acute Physiology and Chronic Health Evaluation II scale (APACHE II). The severity of the aSAH was evaluated with the Hunt and Hess (H–H) scale, and the extent of bleeding was graded using the Fisher scale. Decisions on surgical clipping or endovascular coiling of the ruptured aneurysm were based on the patient’s condition and medical indications and performed within 24 h after the initial bleeding. A control CT scan was performed within 24 h after aneurysm clipping or coiling.

The treatment algorithm was standardised and included euvolemia, analgesia, sedation and catecholamines support when indicated. Patients were mechanically ventilated (bilevel positive airway pressure respiration with controlled respiratory mode, BiPAP) or received passive oxygen therapy. During ICU follow-up, normovolemia and mean arterial pressure (MAP) were maintained above 80 mm Hg and the systolic pressure was 160 to 180 mm Hg. CVP value was maintained between 5 and 8 mm Hg. Patient’s volume status was determined based on central venous pressure (CVP) value, heart rate (HR), arterial blood pressure (ABP), fluid balance, and urine output. In the group of patients with cerebral vasospasm (CV) and those with delayed cerebral ischemia (DCI), induced hypertension was used to improve cerebral circulation. In this group of patients, norepinephrine was used as a first choice treatment due to its combination of α-adrenergic and β-adrenergic receptors stimulation, and the resulting reliable haemodynamic response [15]. The improvement of cardiac output was the second-line treatment following optimization of arterial pressure; thus, milrinone, a selective phosphodiesterase III inhibitor, was used. Isotonic fluids were used to correct hypovolemia and to maintain the euvolemic state. Nimodipine was administered to all patients with aSAH to improve neurological outcome [16].

Neurological examinations were performed by intensivists daily to detect neurological impairment (movement disorders, mental disorders, aphasia). Outcome was assessed during the patient’s stay in the ICU and then in the Neurosurgery Unit. A good short-term outcome was classified as 3–5 and poor as 1–2 in the Glasgow Outcome Scale (GOS). The CV was defined as the mean cerebral blood flow velocity (CBFV) in the middle cerebral artery (MCA) exceeding 120 cm/s or a daily increase of CBFV of ~ 20%, when measured using Transcranial Doppler ultrasonography (TCD) [17]. The ‘ipsilateral’ side was defined as the side of the vasospasm (if occurring) or the side of the aneurysm (in patients without vasospasm). A DCI was defined as a new focal or global neurological impairment that lasted for at least 1 h and cerebral infraction, not caused by other factors (e.g. systemic complications, surgical complications, etc.) [18].

### Regional cerebral desaturation

**Fig. 1:**
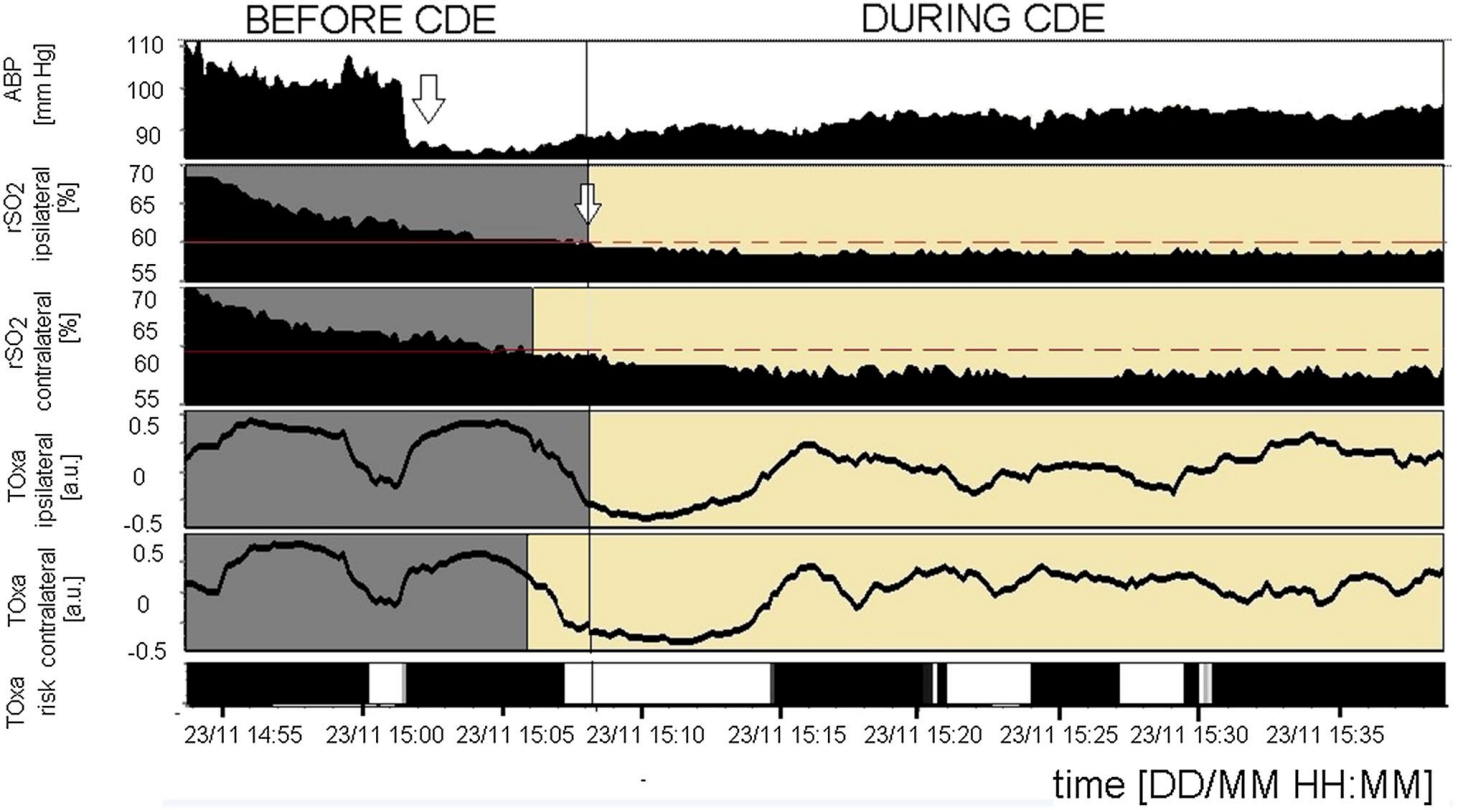
The exemplary time trend of regional cerebral saturation (rSO_2_) on the ipsilateral side in a 61 year old woman with aSAH. The following time-related parameters were defined: the number of cerebral desaturation episodes (CDE_n_), the total time of cerebral desaturation episodes (CDE_t_), and the time of the longest cerebral desaturation episodes (CDE_m_)

A regional cerebral desaturation episode (CDE) [19] was defined as rSO_2_ < 60% for at least 30 min duration [20, 21]. A patient who had at least one CDE during continuous monitoring was classified as having deficient regional cerebral saturation. We introduced the following parameters to describe the CDEs which occurred during NIRS-based continuous rSO_2_ monitoring: the total number of cerebral desaturation episodes (CDE_n_), the total time of the cerebral desaturation episodes (CDE_t_), and the time of the longest cerebral desaturation episode (CDE_m_). The exemplary time trend of rSO_2_ for the ipsilateral side with definitions of CDE_n_, CDE_t_, and CDE_m_ is presented in Fig. 1.

### Cerebral autoregulation

Cerebral autoregulation (CA) was analysed using multimodal monitoring of slow-wave oscillations and assessed using the tissue oxygenation index (TOxa). The TOxa was calculated as a moving linear correlation coefficient between signals of arterial blood pressure (ABP) and rSO_2_. A pressure reactivity index (PRx) was defined as the moving linear correlation coefficient between signals of ABP and ICP. Each of these indices was calculated in overlapping periods of 300 s and updated every 60 s [9]. A disturbed CA was defined as a median ipsilateral or contralateral TOxa above 0 [a.u.] [22].

### Data acquisition

Signal monitoring was performed according to current guidelines [23], started in the ICU within the first 24 h after initial bleeding and continued until the patient’s discharge or when medically required. The rSO_2_ was monitored using an NIRS monitor (Casmed Fore-Sight monitor, Branford, Connecticut, The United States). The probe holder was positioned over the frontal lobe. A pressure transducer (Argon Standalone DTX Plus™, Argon Medical Devices Inc. Plano, TX, USA) was used to invasively measure ABP in the radial/femoral artery. The CBFV in the MCA was collected using a TCD with a 2 MHz probe (Doppler BoxX, DWL Compumedics Germany GmbH, Singed, Germany). The CBFV was monitored, when medically required, for a half an hour per day and the measurements were repeated at least two times in subsequent days. The systolic value of the CBFV was defined as the maximum value of the CBFV during a single cardiac cycle. The ICP was collected using intraparenchymal fibre-optic and piezoelectric ICP sensors (Codman ICP Monitoring System, Johnson & Johnson Professional, Raynham, MA, USA) in patients after surgical clipping. In the group of patients with induced hypertension or inotropic support, haemodynamic parameters of the cardiovascular system were monitored using FloTrac (Edwards Lifesciences Irvine, CA USA), including the following: cardiac output (CO), cardiac index (CI), stroke volume (SV), and variability of stroke volume (SVV). Since the above signals were recorded during monitoring in limited group of patients, they were not analysed in this study. Data acquisition was performed with 200 Hz frequency using the Intensive Care Monitor System (Cambridge Enterprise Ltd, Cambridge, UK). All artefacts were selected manually or by using custom-written algorithms, and further analysis was performed on the representative part of the signals with Matlab® software (MathWorks®, Natick, Massachusetts, USA).

### Statistics

Statistical analysis was performed using STATISTICA version 12 (StatSoft, Inc., Tulsa, USA). Since the hypothesis of normality was rejected based on the Shapiro–Wilk test with the Lilliefors correction for most of the analysed variables, non-parametric tests were applied. Differences in the parameters between the two groups, categorised by any dichotomised criteria defined in this study, were tested using the U Mann–Whitney test (median values) or Fisher’s exact test for the contingency table (number of subjects). A logistic regression model, which was built using backward stepwise elimination, was used to predict mortality risk. The significance of the model was assessed using the following statistics: Likelihood-Ratio test, the value of R^2^ Cox-Snell and R^2^ Negelkerke. The Area Under the Curve (ROC) and the Hosmer–Lemeshow test were applied to determine the goodness of the model’s fit (p-value > 0.05 indicating no evidence of poor fit). The results are presented as medians (interquartile ranges (IQR), 25th–75th percentile).

## Results

### Patient characteristics and signal monitoring

There were 38 patients retrospectively enrolled to this study, where 66% of the subjects were women. The median age was 57 ± 22 (min:29, max:83) [years]. The median H–H score was 3 and the GCS score was 12. The clinical characteristics of patients are presented in Table 1. In 29 patients (76% of the total group), aneurysms were located in the supratentorial region of the brain, whereas nine subjects (24% of the total) had aneurysms in the vertebra-basilar circulation. Nine patients died (24%) and 10 patients (26%) had poor short-term outcome. CV was diagnosed in 14 patients (37% of the entire group) on mean 4 ± 3 day after onset (min 1 day, max 7 day), whereas DCI was found in 13 subjects (34% of the total group). The ABP and rSO_2_ signals were monitored in all patients (n = 38, 100%). The ICP was recorded in 17 subjects (45% of the total group) and the CBFV was monitored in 29 (76% of the total group). The mean time of signal monitoring was 7 ± 4 days (min. 1 day, max. 16 days). The median values of the monitored parameters and CA indices from the whole monitoring time are presented in Supplementary Table 1.

**Table 1:** Patient characteristics in the total group of aSAH patients with and without cerebral desaturation episodes (CDEs)

Parameter	Total group (N = 38)	CDEs (N = 17)	Non-CDEs (N = 21)	p-value
Age (years)	57 ± 22	57 ± 19	56 ± 20	n.s.*
Gender (women), n	25	12 (71%)	13 (62%)	n.s.#
Risk factors				
Hypertension, n	18 (47%)	9 (53%)	9 (43%)	n.s.#
Smoking, n	23 (61%)	10 (59%)	1 (5%)	n.s.#
Other, n	4 (11%)	1 (6%)	3 (14%)	n.s.#
None, n	2 (5%)	1 (6%)	1 (5%)	n.s.#
Clinical features				
Apache II grade	14	16	11	n.s.*
GCS grade	12	13	14	n.s.*
FOUR, grade	13	15	16	n.s.*
H–H grade	3	4	2	0.016*
Grade 1, n	7 (18.5%)	1 (6%)	6 (29%)	n.s.#
Grade 2, n	7 (18.5%)	1 (6%)	6 (29%)	n.s #
Grade 3, n	8 (21%)	6 (35%)	2 (9%)	n.s #
Grade 4, n	8 (21%)	3 (18%)	5 (24%)	n.s #
Grade 5, n	8 (21%)	6 (35%)	2 (9%)	n.s #
Fisher grade	3	4	3	n.s.*
Grade 2, n	8 (21%)	3 (18%)	5 (24%)	n.s.#
Grade 3, n	12 (32%)	4 (24%)	8 (38%)	n.s.#
Grade 4, n	18 (47%)	10 (58%)	8 (38%)	n.s.#
Therapeutic intervention				
Clipping, n	16 (42%)	9 (53%)	7 (41%)	n.s.#
Coiling, n	19 (50%)	6 (35%)	13 (62%)	n.s.#
Conservative treatment, n	3 (8%)	2 (12%)	1 (5%)	n.s.#
Outcome and complications				
Neurological disorders at discharge, n	16 (42%)	8 (47%)	9 (43%)	n.s.#
DCI, n	13 (34%)	6 (35%)	7 (33%)	n.s.#
CV, n	14 (37%)	6 (35%)	8 (38%)	n.s.#
GOS score at discharge	4	3	5	0.013*
Poor short-term outcome, n	10 (26%)	7 (41%)	3 (14%)	n.s.#
Mortality, n	9 (27%)	6 (35%)	3 (14%)	n.s.#

Data are presented as median ± interquartile range or as the number of subjects (% of the group)

*APACHE II* acute physiology and chronic health evaluation II, *GCS* glasgow coma scale, *FOUR* full outline of unresponsiveness scale, *H*–*H* hunt and hess scale, *EVD* external ventricular drainage, *DCI* delayed cerebral ischemia, *CV* cerebral vasospasm, *GOS* glasgow outcome scale

p-value refers to the results of the U Mann–Whitney test (marked as *) or Fisher’s exact test for the contingency table (marked as #)

Statistical significant values were marked in bold

### Cerebral desaturation episodes

**Fig. 2:**
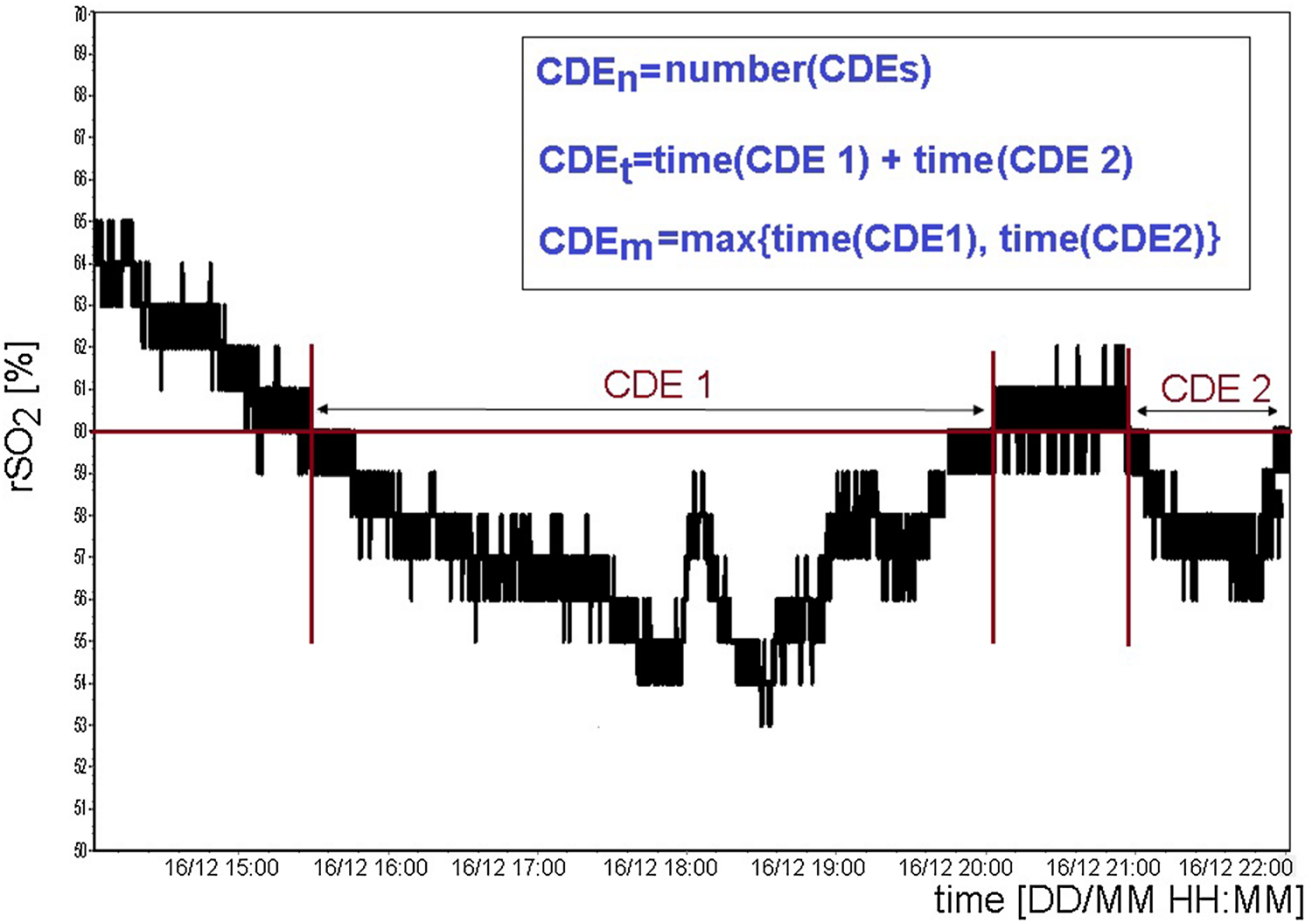
The cerebral desaturation episode (CDE) on the ipsilateral side in a 41 year old woman with aSAH. The decrease in rSO_2_ was related to worse cerebral autoregulation and was preceded by a drop in ABP. *ABP* arterial blood pressure, *rSO*_*2*_ regional cerebral oxygen saturation, *TOxa* tissue oxygenation index. In the Toxa risk panel disorders of cerebral autoregulation (TOxa > 0) are marked in black. The arrows indicate the starting point of a decrease in the signals

CDEs occurred in 17 patients (44% of the total group) of which 7 patients had CDEs on both sides of the brain and 10 patients had CDEs only on one side (4 patients on the ipsilateral side, 6 on the contralateral side). In the group of patients with CDEs, 6 died, which is 67% of the total cases that resulted in death. The exemplary time trends of the monitored signals during the CDEs on both sides are presented in Fig. 2. Median values of the ipsilateral (p = 0.02) and contralateral (p = 0.01) rSO_2_ were significantly lower in the group with CDEs than the respective values in the non-CDE group, see Supplementary Table 1. Comparisons of the clinical parameters in the groups with and without CDEs are presented in Table 1. There was a significant difference in the H–H grades for patients with or without a CDE (4 scores vs. 2 scores, p = 0.016). Furthermore, patients with a CDE had a worse short-term outcome in the GOS scale (median GOS grade 3) than patients without a CDE (median GOS grade 5), p = 0.013.

### Cerebral desaturation episodes versus cerebral autoregulation

We found that ipsilateral TOxa was disordered in 27 patients (71%) and contralateral TOxa was impaired in 26 patients (68%) of the total group. In the analysed cohort 7 out of 9 cases resulting in death (78%) were in patients who suffered from a disturbed CA. Disturbances in CA during a CDE were found in 6 patients ipsilaterally (6/11; 55%) and 10 contralaterally (10/13; 77%). In most of the analysed subjects, the disturbances in CA were found both before and during the CDEs (see Fig. 2). PRx was calculated only in 6 patients with a CDE (6/17; 35%); however, we observed that the values of PRx corresponded with the respective TOxa values and indicated a disturbed CA: median PRx during the CDEs: 0.284 (0.157–0.489) [a.u.].

### Parameters of CDEs

In the entire group, the CDE_n_ was 3 for the ipsilateral side and 4 for the contralateral side. The median values of CDE_n_, CDE_t_, and CDE_m_ in aSAH patients, categorized by the severity of the haemorrhage and short-term outcome, are shown in Table 2. Patients with severe haemorrhage in the H–H scale had a longer CDE_t_ on the contralateral side than the ones with moderate haemorrhage [h:min]: 8:15 (6:26–8:55) versus 1:23 (1:19–4:17), p = 0.038. Furthermore, patients with severe aSAH had a significantly longer CDE_m_ on the contralateral side than ones with moderate aSAH [h:min]: 2:05 (2:04–5:20) versus 0:49 (0:44–2:12) p = 0.038. We observed that patients with poor short-term outcome had a longer CDE_m_ on the ipsilateral side than ones with good short-term outcome [h:min]: 5:43 (3:04–9:35) versus 1:47 (0:41–2:10), p = 0.018. According to the ROC curves for predicting a poor short-term outcome, the cut-off value for the CDE_m_ on the contralateral side was 2:05[h:min] (Z = 2.026, p = 0.042, AUC = 0.718) and the cut-off value for a CDE_t_ on the contralateral side was 8:15[h:min] (Z = 2.065, p = 0.039, AUC = 0.721).

**Table 2:** Parameters of cerebral desaturation episodes (CDEs) calculated in the total group of aSAH patients, and in patients with good/poor short term-outcome and with severe/moderate aSAH

Parameter	Total group (n = 17)	Moderate aSAH (n = 10)	Severe aSAH (n = 7)	p-value	Good outcome (n = 10)	Poor outcome (n = 7)	p-value
CDE_n_ ipsilateral [a.u.]	3 (1–4)	3 (1–4)	1 (1–2)	0.132	3 (1–4)	2 (1–3)	0.483
CDE_n_ contralateral [a.u.]	4 (2–7)	2 (2–4)	6 (3–12)	0.195	5 (2–7)	7 (2–12)	0.565
CDE_t_ ipsilateral [h:min]	4:18 (2:40–6:13)	4:02 (2:47–5:07)	6:57 (1:41–12:20)	0.770	2:55 (0.41–4:13)	8:43 (4:27–12:20)	0.108
CDE_t_ contralateral [h:min]	6:26 (1:28–10:35)	1:23 (1:19–4:17)	8:15 (6:26–8:55)	**0.038**	3:54 (1:19–6:26)	9:44 (8:15–10:35)	0.074
CDE_m_ ipsilateral [h:min]	2:10 (1:25–3:28)	2:06 (1:25–2:46)	5:20 (1:41–9:35)	0.298	1:47 (0.41–2:10)	5:43 (3:04–9:35)	**0.018**
CDE_m_ contralateral [h:min]	2:14 (0:52–5:04)	0:49 (0.44–2:12)	2:05 (2:04–5:20)	**0.038**	1:53 (0:46–2:12)	5:11 (2:05–10:35)	0.100

*p*-values less than 0.05 are marked in bold

*CDE*_*n*_ the total number of cerebral desaturation episodes, *CDE*_*t*_ the total time of cerebral desaturation episodes, *CDE*_*m*_ the time of the longest cerebral desaturation episode; the differences were tested using the U Mann–Whitney test

### Multiple logistic regression model to predict short-term mortality

The following parameters were used as predictors of poor short-term outcome in a multiple linear regression model: Fisher grades, Apache II grades, median ABP, median ipsilateral and contralateral rSO_2_, median ipsilateral and contralateral TOxa, occurrence of CA disorders, CDE_m_ and CDE_t_ on the contralateral side, dichotomised based on ROC curve thresholds for a poor short-term outcome. Among all the possible subsets of predictors, the model with the highest likelihood based on maximum likelihood estimation was chosen. The poor short-term outcome logistic regression model included the extent of haemorrhage on the Fisher scale, median ABP, and CDE_m_ on the contralateral side as a binary variable (see Table 3). The model was statistically significant: Likelihood–Ratio test: 18.13, p = 0.0004, R^2^ Cox–Snell: 0.38, R^2^ Negelkerke: 0.55, Hosmer–Lemeshow test: 6.85, p = 0.55. The area under the ROC Curve was 0.868 ± 0.071, showing that the model had good discrimination ability.

**Table 3:** The binary logistic regression model for predicting poor short-term outcome in 38 patients with diagnosed aneurysmal subarachnoid haemorrhage (aSAH)

Model parameter	Wald’s statistic	p-value	Adj OR	95% CI
Extent of haemorrhage in the Fisher scale [a.u.]	2.56	0.11	3.60	0.75–17.34
Mean ABP [mm Hg]	3.18	0.07	3.60	0.79–1.01
CDE_m_ for contralateral side*	4.14	0.04	15.39	1.11–214.16

*ABP* arterial blood pressure, *CDE*_*m*_ the time of the longest cerebral desaturation episode (CDE) expressed as a binary variable regarding the critical threshold

*According to the ROC curve the threshold value for CDE_m_ for the contralateral side as a predictor of poor-short term outcome was 2.08 [h] (Z = 2.026, p = 0.042, AUC = 0.718)

## Discussion

Our results indicate that the time-related parameters of CDEs, namely: the total time of CDEs (CDE_t_) and the time of the longest CDE (CDE_m_) were longer in patients with severe aSAH, classified by the H–H scale. Moreover, a longer CDE_m_ was related to poor short-term outcome.

It has been shown in previous studies that long cerebral desaturation events during cardiosurgical operations may lead to increased morbidity, mortality [24], neurocognitive disorders [25] or postoperative cognitive dysfunction [26]. The duration of the ischemia is a critical determinant of tissue damage, thus only a CDE which exceeds a time threshold may have a significant impact on brain condition, cause neurological disorders or lead to a worse outcome. In our study, the threshold value of a CDE_m_ on the contralateral side was 2 h 5 min. This result is consistent with previous studies. It has been shown in research on pigs that the time of a CDE should be at least 2 h to result in neurological injury [24]. However, another analysis of 265 patients after coronary artery bypass grafting, revealed that a CDE lasting at least 50 min could result in cognitive decline or a longer hospital stay [25]. Thus, the critical threshold of the CDE time requires further investigation in a large cohort of aSAH patients.

Our results also indicated that CDEs were longer and occurred more often in patients with severe aSAH, contrary to the ones with moderate aSAH. It is noteworthy that patients with severe aSAH often have extended haemorrhage, which directly impacts cerebral blood flow and cerebral metabolic rate. Cerebral oxygenation could be reduced as the direct neurotoxic result of subarachnoid blood and caspase-dependent and caspase-independent death receptor elevation [26]. Furthermore, insufficient cerebral saturation could be the result of ischemic damage or reperfusion injury or a reduced metabolic rate which occurred in the first short acute phase of aSAH [3]. These findings imply that NIRS monitoring could be beneficial in patients classified as 4–5 in the H–H scale.

In our study, the ipsilateral side and contralateral side were defined to differentiate the side of the brain affected by the aneurysm and CV. However, the results showed that changes in cerebral oxygenation were not restricted to the territory of the ruptured aneurysm or CV, but also involved other areas of the brain. In the acute phase of aSAH when the early brain injury occurred, signalling cascades initiated a blood–brain barrier disturbance, inflammatory activation, and oxidative stress in areas of the brain distant from the aneurysm location.

CA impairment, actuated by the hemodynamic discrepancy between neurons and vessels and then contributing to reduced CBF, is commonly observed in aSAH [3]. We found that the CA was disturbed in about 70% of the total group of aSAH patients and accompanied the occurrence of a CDE. This observation is in line with previous studies, which showed that CA, assessed using TOxa was impaired after aSAH [27]. Moreover, we found that cerebrovascular autoregulation, evaluated using the PRx index, was impaired during CDEs. This observation is in line with another study which showed that the PRx index correlated with a state of CBF and CMRO_2_, which was determined using PET [28].

The multiple logistic regression model with the highest likelihood based on MLE showed that a CDE_m_ above the critical threshold, the extent of haemorrhage in the Fisher scale and mean arterial blood pressure were predictors of a poor short-term outcome. However, only CDE_m_ on the contralateral side was statistically significant as parameter (W = 4.14; p = 0.04; OR = 15.39) in the logistic regression model and the interpretation concerning the rest of the parameters (median ABP and the extent of haemorrhage on the Fisher scale) should be limited. The CDE_m_ above the critical threshold was the best predictor of short-term outcome after aSAH, which suggests that the monitoring of the time-related parameters of CDEs may have beneficial prognostic implications. The observation that the higher ABP may lead to worse short-term outcome is related with the ineffectiveness of induced hypertension (one of the triple-H therapy, which was applied in our patients with cerebral vasospasm and these with delayed cerebral ischemia in order to improve cerebral circulation). One of the latest studies has shown that this treatment could be insufficient in aSAH patients with symptomatic vasoconstriction and elevated initial troponin I level, indicating neurogenic damage to the heart [29]. The Fisher grading has been shown to be a predictor of outcome in a previous study as related to a higher risk of vasospasm or delayed cerebral ischemia [30].

Moreover, there is no clearly defined clinical way of treating aSAH patients in ICU, and applied treatments are subject to constant criticism. ICP monitoring undoubtedly helps in maintaining the desired brain perfusion pressure, but is an invasive method and has its limitations. Transcranial Doppler is effective in detecting the contraction of the large vessels, but we monitor patient for a short period of time (about 30 min). What happens to the patient during the remaining time, especially if they are intubated and require administering of sedative drugs remains an interesting issue. Data on this subject is very limited and there are no studies showing that the use of NIRS may improve the outcome in adult neurocritical patients. NIRS could be successfully applied to monitor regional cerebral oxygenation and CA in patients with aSAH for a long period of time [28] since it is easy to set up and maintain as well as cost-effective. NIRS could constitute a part of a multimodality neuromonitoring in neurocritical care, thus it should be interpreted with other systemic variables (PaO_2_, PaCO_2_, FiO_2_, mean arterial blood pressure) and brain-specific variables (CBF, cerebral vasospasm, cerebral autoregulation or brain tissue gradients for oxygen diffusion) [31].

The controversy about using NIRS in clinical practise has been related to the lack of a standard measurement protocol, from using different types of devices with a variety of implemented algorithms. Moreover, external factors such as cerebrospinal fluids or blood in the subarachnoid or subdural space may have an effect on the measurements [9, 30]. Moreover, it has been shown that NIRS can monitor normal cerebral oxygenation values in patients with brain death [32, 11, 33]. It has been hypothesized that normal rSO2 during prolonged cardiac arrest may be the result of the absence of cerebral metabolism, preservation of the extracranial circulation (NIRS device is significantly influenced by the oxygenation of the scalp and skull), or a maintained residual cerebral perfusion. There were 9 cases of death in our cohort, whereas rCD episodes occurred in 6 cases of death. Although we did not monitor signals up to the moment of patient’s death, we observed that the values of rSO_2_ gradually decreased in 4 out of 6 cases of death and rCD episodes, accompanied by a significant and long-term impairment of the cerebral autoregulation.

However, there are several limitations to this analysis. Firstly, there are a lot of factors related to the treatment of a serious condition which may affect the monitored signals and parameters, even though they represent the real critical care environment: analgesia, sedatives, intravenous anaesthetics, and nursery manoeuvres [32]. Moreover, as it has been shown in previous research, the value of rSO_2_ may be influenced by the depth and type of administered anaesthesia, the systemic arterial blood pressure, haematocrit, arterial carbon dioxide partial pressure (P_a_CO_2_), arterial oxygen saturation (SaO_2_), pH and temperature [33, 34]. In our study, the physiological factors which could have had an effect on the rSO_2_, were maintained in the recommended range for aSAH patients [14]. Furthermore, the entire group was drug-homogenous. Since all patients had extensive monitoring of systemic oxygenation, CO2, and hemoglobine levels and these values were maintained within normal range, we assumed that rSO2 changes result from disturbances in the circulation of the brain. Theoretically, the use of induced hypertension or improvement of cardiac output should improve rSO2; however, we did not notice improvement in all patients. In our study, CBFV signals were used to determine the cerebral vasospasm and were not measured routinely, but when required. Because CBFV was not monitored during the CDEs in the entire group we decided not to analyse the CBFV-based mean velocity index Mxa. Furthermore, it should be noted that an ICP sensor was introduced to the intracranial compartment during operation (surgical clipping of the aneurysm) and was not routinely implemented in aSAH patients, but when medically necessary in a fraction of the analysed group, thus we did not determine the PRx in the entire group of patients. In various clinical practices, cerebral desaturation episodes are defined using different thresholds for NIRS-based rSO_2_ and time of duration. In a recent study about hemodynamic instability in the postoperative period following cardiac surgery, a CDE was described as rSO_2_ of less than 60% for at least 60 s [19]. In other research, rSO_2_ of 60% to 65% was suggested as the threshold value [34] for a CDE after thoracic aortic surgery. Even lower values of cerebral saturation were proposed in one definition of a CDE, namely: rSO_2_ below 50% in coronary artery bypass grafting [25] or rSO_2_ less than 40% in patients undergoing cardiac surgery with cardiopulmonary bypass [35]. The current opinions regarding the system of assessing patients’ outcomes differ among researchers/clinicians. One of the most popular scales used to assess the long-term outcome is modified Rankin scale (mRS). However, in our study, we focused on the early results of the treatment, thus GOS scale was more adequate to assess the outcome [36, 37]. Nevertheless, other clinical scale to assess patients’ long-term outcome needs to be applied in further research concerning relationship between the regional cerebral desaturation episodes and outcome in aSAH patients. Finally, the analysed data comes from patients hospitalised between 2015 and 2017. Thus, the standards of post-operative care could have slightly changed in the past few years. The group of aSAH patients which were analysed in this study was limited and came from one medical centre. Further research is warranted to confirm and better evaluate the observations presented in this paper.

## Conclusion

We have demonstrated that time-related parameters describing CDEs are related to short-term outcome and severity of aSAH. Our findings suggest that NIRS can be used as a valuable part of a bedside neuromonitoring and may provide additional information about regional cerebral oxygenation and cerebral autoregulation. The more we know about SAH-induced cerebral desaturation, the more efficient treatment strategies can be developed in aSAH patients. However, many questions about the clinical usefulness of the NIRS still remain open for discussion.

## Electronic supplementary material

Below is the link to the electronic supplementary material.

Supplementary material 1 (DOCX 13 kb)
