# Supplementary material for: The relationship between the time of cerebral desaturation episodes and outcome in aneurysmal subarachnoid haemorrhage: a preliminary study
Source: J Clin Monit Comput. 2019 Aug 20;34(4):705–14. doi: 10.1007/s10877-019-00377-x (PMC7367903; doi:10.1007/s10877-019-00377-x)
Supplement: Supplementary file 1 — Supplementary material 1 (DOCX 13 kb) [file 10877_2019_377_MOESM1_ESM.docx]

**Supplemental Material**

**Supplemental Table 1** Median values (upper quartiles–lower quartiles) of monitored signals and cerebral autoregulation indices in the total group of aneurysmal subarachnoid haemorrhage (aSAH) patients with and without cerebral desaturation episodes (CDEs) throughout the total monitoring time.

| **Parameter** | **Total group** | **with CDEs** | **without CDEs** | **p-value** |
| --- | --- | --- | --- | --- |
| ABP  [mmHg] | n=38  90.15 (83.99–95.36) | n=17  92.92 (81.59–95.36) | n=21  89.21 (84.42–95.33) | n.s. |
| rSO_2_  ipsilateral  [%] | n=38  70.39 (67.20–74.99) | n=17  69.00 (66.19–71.00) | n=21  73.00 (68.00–76.99) | **0.02** |
| rSO_2_  contralateral  [%] | n=38  71.00 (66.43–73.80) | n=17  68.27 (64.00–71.95) | n=21  72.44 (69.76–74.36) | **0.01** |
| ICP  [mm Hg] | n=17  7.88 (5.21–13.90) | n=9  7.88 (6.17–18.17) | n=8  9.32 (5.18–12.48) | n.s. |
| CBFV  systolic ipsilateral [cm/s] | n=29  75.15 (58.48–94.17) | n=14  86.30 (58.48–96.16) | n=15  74.75 (53.22–94.17) | n.s. |
| CBFV  systolic  contralateral [cm/s] | n=29  73.77 (52.42–103.95) | n=14  80.71 (54.41–102.20) | n=15  73.07 (46.79–109.85) | n.s. |
| TOxa  ipsilateral  [a.u.] | n=38  0.04 (–0.02–0.10) | n=17  0.03 (-0.01–0.05) | n=21  0.05 (-0.02–0.10) | n.s. |
| TOxa  contralateral  [a.u.] | n=38  0.04 (0.00–0.10) | n=17  0.04 (-0.02–0.06) | n=21  0.04 (0.01–0.10) | n.s. |
| PRx  [a.u.] | n=14  0.10 (–0.01–0.30) | n=9  0.17 (0.08–0.51) | n=8  0.03 (-0.02–0.30) | n.s. |

**Abbreviation:** ABP – arterial blood pressure, rSO_2_ – regional cerebral oxygen saturation, ICP – intracranial pressure, CBFV systolic – cerebral blood flow velocity systolic, TOxa – tissue oxygenation index, PRx – pressure reactivity index; the differences between patients with and without regional cerebral desaturation episodes were tested using the U Mann-Whitney test.
